# Adjuvant chemotherapy or no adjuvant chemotherapy? A prediction model for the risk stratification of recurrence or metastasis of nasopharyngeal carcinoma combining MRI radiomics with clinical factors

**DOI:** 10.1371/journal.pone.0287031

**Published:** 2023-09-26

**Authors:** Qiaoyuan Wu, Yonghu Chang, Cheng Yang, Heng Liu, Fang Chen, Hui Dong, Cheng Chen, Qing Luo

**Affiliations:** 1 The Public Experimental Center of Medicine, Department of Pathology, Affiliated Hospital of Zunyi Medical University, Zunyi, Guizhou, P. R. China; 2 School of Medical Information Engineering of Zunyi Medical University, Zunyi Medical University, Zunyi, Guizhou, P. R. China; 3 The Third Clinical Medical College of Ningxia Medical University, Yinchuan, Ningxia, P. R. China; 4 Department of Radiology, Affiliated Hospital of Zunyi Medical University, Zunyi, Guizhou, P. R. China; 5 Department of Thoracic Surgery, Affiliated Hospital of Zunyi Medical University, Zunyi, Guizhou, P.R. China; Xijing Hospital, Air Force Medical University, CHINA

## Abstract

**Background:**

Dose adjuvant chemotherapy (AC) should be offered in nasopharyngeal carcinoma (NPC) patients? Different guidelines provided the different recommendations.

**Methods:**

In this retrospective study, a total of 140 patients were enrolled and followed for 3 years, with 24 clinical features being collected. The imaging features on the enhanced-MRI sequence were extracted by using PyRadiomics platform. The pearson correlation coefficient and the random forest was used to filter the features associated with recurrence or metastasis. A clinical-radiomics model (CRM) was constructed by the Cox multivariable analysis in training cohort, and was validated in validation cohort. All patients were divided into high- and low-risk groups through the median Rad-score of the model. The Kaplan-Meier survival curves were used to compare the 3-year recurrence or metastasis free rate (RMFR) of patients with or without AC in high- and low-groups.

**Results:**

In total, 960 imaging features were extracted. A CRM was constructed from nine features (seven imaging features and two clinical factors). In the training cohort, the area under curve (AUC) of CRM for 3-year RMFR was 0.872 (P <0.001), and the sensitivity and specificity were 0.935 and 0.672, respectively; In the validation cohort, the AUC was 0.864 (P <0.001), and the sensitivity and specificity were 1.00 and 0.75, respectively. Kaplan-Meier curve showed that the 3-year RMFR and 3-year cancer specific survival (CSS) rate in the high-risk group were significantly lower than those in the low-risk group (P <0.001). In the high-risk group, patients who received AC had greater 3-year RMFR than those who did not receive AC (78.6% vs. 48.1%) (p = 0.03).

**Conclusion:**

Considering increasing RMFR, a prediction model for NPC based on two clinical factors and seven imaging features suggested the AC needs to be added to patients in the high-risk group and not in the low-risk group.

## 1. Introduction

Nasopharyngeal carcinoma (NPC) is a malignant tumour of the head and neck originating from the nasopharyngeal mucosal lining with uneven endemic distribution [1]. According to the 2020 Global Cancer Statistics, the new incidence and new mortality of NPC accounted for 0.7% and 0.8% of all cancers, respectively [2].

At present, tumour node-metastasis (TNM) stage is one of the most important factors in predicting the prognosis of NPC. However, the prognosis of patients with the same TNM stage receiving similar treatment varies greatly. 20%-30% patients still experienced recurrence or metastasis, resulting in poor prognosis [3, 4]. This phenomenon may be explained by this fact that the TNM staging system mainly reflects the degree of invasion of the tumour anatomical structure and cannot accurately reflect the heterogeneity within the tumour.

Adjuvant chemotherapy (AC) refers to chemotherapy performed after radical local treatment (surgery or radiotherapy) to prevent the recurrence or metastasis of micrometastatic lesions that may exist. Chen et al. found that AC did not improve failure-free survival after concurrent chemoradiotherapy (CCRT) in locoregionally advanced nasopharyngeal carcinoma (LA-NPC) [5]. Therefore, for patients with LA-NPC, CSCO recommends induction chemotherapy (IC) combined with concurrent radiotherapy (CCRT) as category IA and CCRT combined with AC as category IB [6]. However, The National Comprehensive Cancer Network (NCCN) and European Society for Medical Oncology (ESMO) guidelines recommend that IC and AC have equal status [7, 8]. Does AC benefit patients? In order to solve the problem, many scholars stratified NPC patients and found that AC was suitable for some specific populations and improved survival, such as N stage stratification, EB virus infection stratification [9–12]. Therefore, A more accurate combined model is necessary to predict the prognosis of NPC and identify patients who may benefit from AC.

Radiomics refers to the extraction and analysis of a large number of advanced quantitative imaging features from medical images [13, 14]. As a new technique, radiomics has been studied for many applications, such as clinical diagnostics, pathological typing, prognosis prediction and clinical decision-making for a variety of cancers, including lung cancer, colon cancer and kidney cancer [15–18]. In many studies, radiomics demonstrated a good predictive ability [19, 20], because it revealed the internal heterogeneity of cancer tissue in terms of cytology, physiology and genetic informatics by extracting features from within and around the tumor [21]. Significant phenotypic differences in tumour region imaging can compensate for spatiotemporal heterogeneity that cannot be elucidated by clinical factors [22–24]

Thus, in this study, a prognostic combined model was constructed based on radiomics and clinical features that could accurately screen for suitable AC patients.

## 2. Methods and materials

### 2.1 Sample size

In the research, nine features were finally retained, and then the estimated sample size was at least 90 cases. For the sample size of the validation cohort, we performed power calculation by PASS, and found that the minimum sample size was 36. In our study, 140 patients (98 in the training cohort and 42 in the validation cohort) were enrolled to ensure that the study was fully analyzed.

### 2.2 Patients

**Fig 1:**
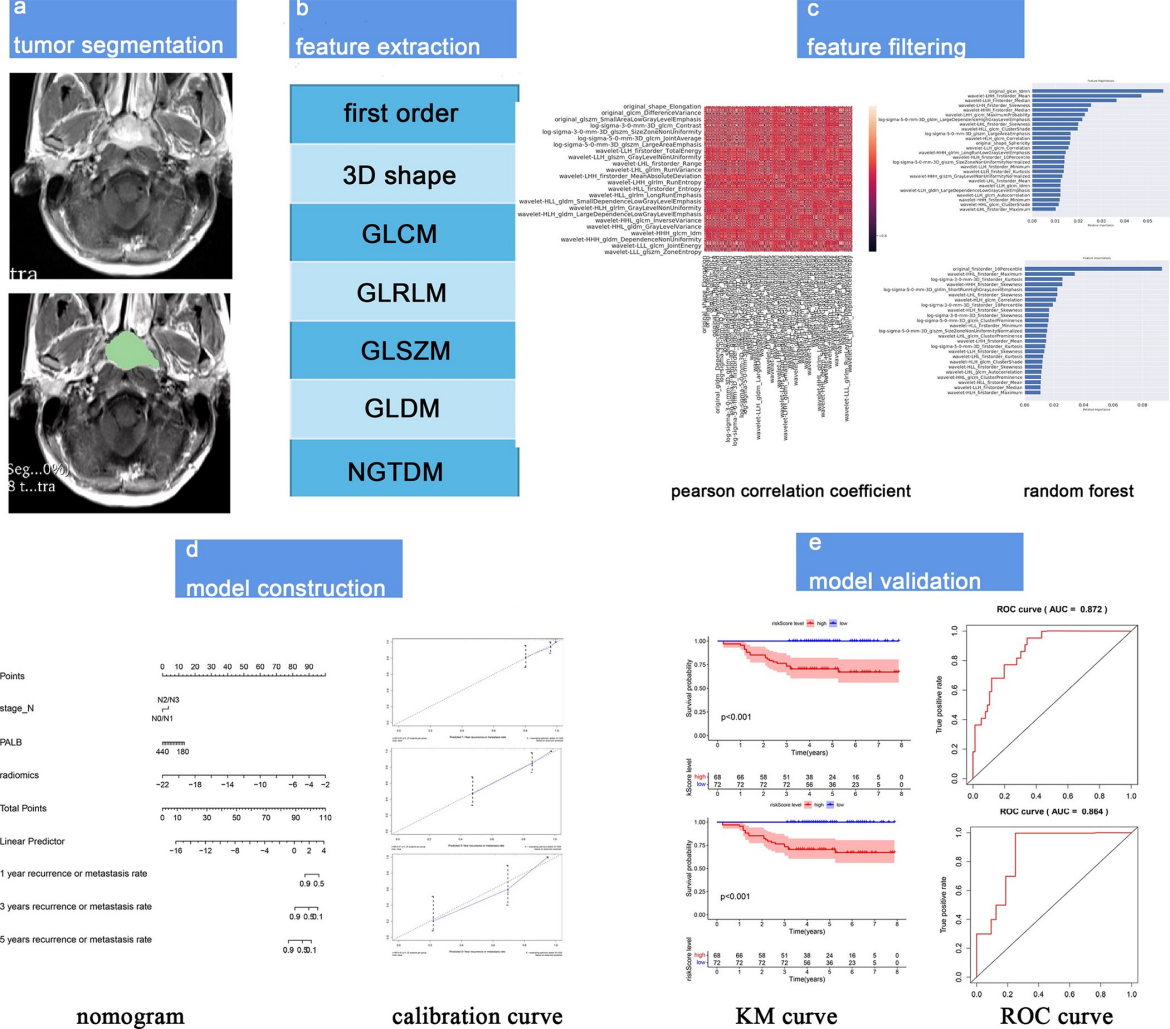
The workflow of radiomics nomogram establishment. a) Tumor segmentation in 3D-slicer; b) Seven types of features were extracted; c) Selection of features by pearson correlation coefficient (PCC) and random forest (RF); d) Radiomics nomogram construction and application; e) Evaluation and validation of models.

This study was approved by the Ethics Committee of the Affiliated Hospital of Zunyi Medical University (Approval No.: KLLY-2020-012). A retrospective analysis was performed for non-metastatic NPC patients newly diagnosed at the Affiliated Hospital of Zunyi Medical University from February 2013 to December 2017. The eligibility criteria were as follows: a) histologically diagnosed undifferentiated, non-keratinized carcinoma; b) examinations were performed to determine staging (such as MRI scan); c) complete clinical data, including age, sex, Epstein-Barr virus DNA (EBV-DNA), and TNM stage (eighth edition of AJCC) were available; d) no other malignancies were present. The exclusion criteria were as follows: a) treatment before baseline MRI scan, such as radiotherapy, chemotherapy, immunotherapy and surgery; b) incomplete clinical data; c) artefacts, blurs, faults, and disordered slices in the MRI; e) MRI examination was performed in another hospital; f) non-standard treatment; g) other deaths except those caused by NPC before the end of follow-up. This workflow is shown in **Fig 1.**

### 2.3 MRI scan

A total of 140 patients received 1.5T head and neck MRI (GE, USA, TR:350ms, TE:10ms, 5mm thickness) at the Affiliated Hospital of Zunyi Medical University, including enhanced-MRI sequence, T1WI sequence, and T2WI Flair sequence.

### 2.4 Follow-up and clinical endpoint

In the first two years of follow-up, patients were examined by routine imaging methods every three months, every six months from the third year to the fifth year, and annually thereafter. The primary endpoint, 3-year recurrence or metastasis free survival (RMFS), defined as the time from the date of the first MRI to the date of recurrence or metastasis or to the date of the follow-up (the follow-up time was over 36 months). The 3-year cancer-specific survival (CSS) was analysed as a secondary endpoint and defined as the time between the date of the first MRI to the date of death due to NPC. If nasopharyngoscopy, head and neck MRI, PET/CT and other examinations mentioned the possibility of metastasis or recurrence, further examination (such as MRI or biopsy) was required to identify potential involved sites. If further examination results were negative, the patients were followed up with every three months for at least one year.

### 2.5 Collection of clinical data

The clinical data include age, sex, T stage, N stage (AJCC 8th edition), family history, ethnicity, treatment scheme, leukocytes, neutrophils, eosinophils, basophils, hemoglobin, red blood cells, platelets, albumin, prealbumin, alanine aminotransferase, aspartate aminotransferase, alkaline phosphatase, serum creatinine, blood urea nitrogen, EBV-DNA, survival status, and time to recurrence or metastasis.

### 2.6 Collection of image data

We obtained DICOM images (including T1WI, T2WI Flair, and enhanced-MRI sequences) directly from the picture archiving and communication system (PACS) system. The enhanced-MRI sequences were imported into "3D slicer" software, in which the whole region of interest (ROI) was drawn slice-by-slice to obtain the 3D segmented image. The tumor boundary was outlined mainly with reference to T2WI Flair and T1WI sequences. All images were segmented by two intermediate physicians with 10 years of experience and then reviewed by two associate chief physician who work in head and neck oncology.

### 2.7 Feature extraction and filtering

Features were extracted from the ROI using the Python “pyradiomics” package (implemented in Python, version 3.6). Image features include original image and filtered derived image.

We used intra- and interclass correlation coefficient (ICC) to assess the effects of variations in manual segmentation on radiomics feature values. ICC values greater than 0.75 indicate good agreement.

In order to avoid model overfitting, the extracted imaging features were filtered through the PCC and RF. PCC was used to assess the correlation between each pair of features. if correlation coefficient was greater than 0.7, one feature was excluded from each pair of correlated features (try to keep the original feature). Finally, we obtained the factors that were most closely associated with recurrence or metastasis.

### 2.8 Model construction

Eligible patients were randomly divided into a training cohort (n = 98) and an independent validation cohort (n = 42) in a ratio of 7:3. Both cohorts were well-balanced in baseline demographics and clinical factors by randomly grouping.

A clinical model (CM) was constructed for predicting the recurrence or metastasis of NPC. 1) Cox univariate analysis was performed by the clinical factors in the training cohort (clinical data included age, sex, EBV-DNA, platelet, ALP, ASP). 2) Construction of a Cox multivariate model with data from the training cohort. 3) Establishing a Receiver Operating Characteristic Curve (ROC) of 3-year recurrence or metastasis free rate (RMFR) to verify the sensitivity and specificity of the model. ROC curves were widely used to assess the sensitivity, specificity and accuracy of models [25, 26].

A radiomics model (RM) was constructed. 1) Cox univariate analysis in the training cohort was performed by the imaging features which selected by the PCC and the RF; 2) RM was constructed by Cox multivariate analysis; 3) A ROC of 3-year RMFR was established.

Cox multivariate regression analysis was performed on the clinical factors and imaging features that were statistically different from those obtained from the Cox univariate regression analysis, to constructed the clinical-radiomics model (CRM), and the Rad-scores were calculated. Similarly, the sensitivity and specificity of the model were verified by the ROC.

### 2.9 Evaluation and validation of models

The prediction models were constructed through the training cohort, and were verified in the verification cohort. We used the Delong test to compare the area under curves (AUCs) of the CRM with those of the CM and the RM respectively. Kaplan-Meier curves were plotted for the CRM with high-and low-risk groups. Log-rank test was used to test the difference of survival curves between high- and low-risk groups. The Rad-score was used to predict the recurrence or metastasis rate of NPC at 1, 3 and 5 years by nomogram, and the prediction efficiency of the model was verified by calibration curves.

### 2.10 Comparison of the survival benefit of patients with or without AC with subgroup analysis

The patients in two cohorts were stratified into high- and low-risk subgroups based on the median Rad-score of CRM. The comparison of the survival benefit of patients with or without AC was performed in the high- and low-risk subgroups by analyzing Kaplan-Meier survival curves.

### 2.11 Statistical analysis

We performed the clinical factors in the training and validation cohorts from the primary dataset using the Fisher’s exact test and χ^2^ tests. the RMFR and the CSS rate between the two groups were compared using log-rank test, and Kaplan-Meier curves were used to provide time-to-event data. All analysis were performed using SPSS 18.0 (https://www.ibm.com/spss), R 3.6.3 (http://www.R-project.org) and Python 3.6 (https://www.python.org/)). Two-sided p values < 0.05 were considered statistically significant.

## 3. Results

### 3.1 Patient characteristics

**Fig 2:**
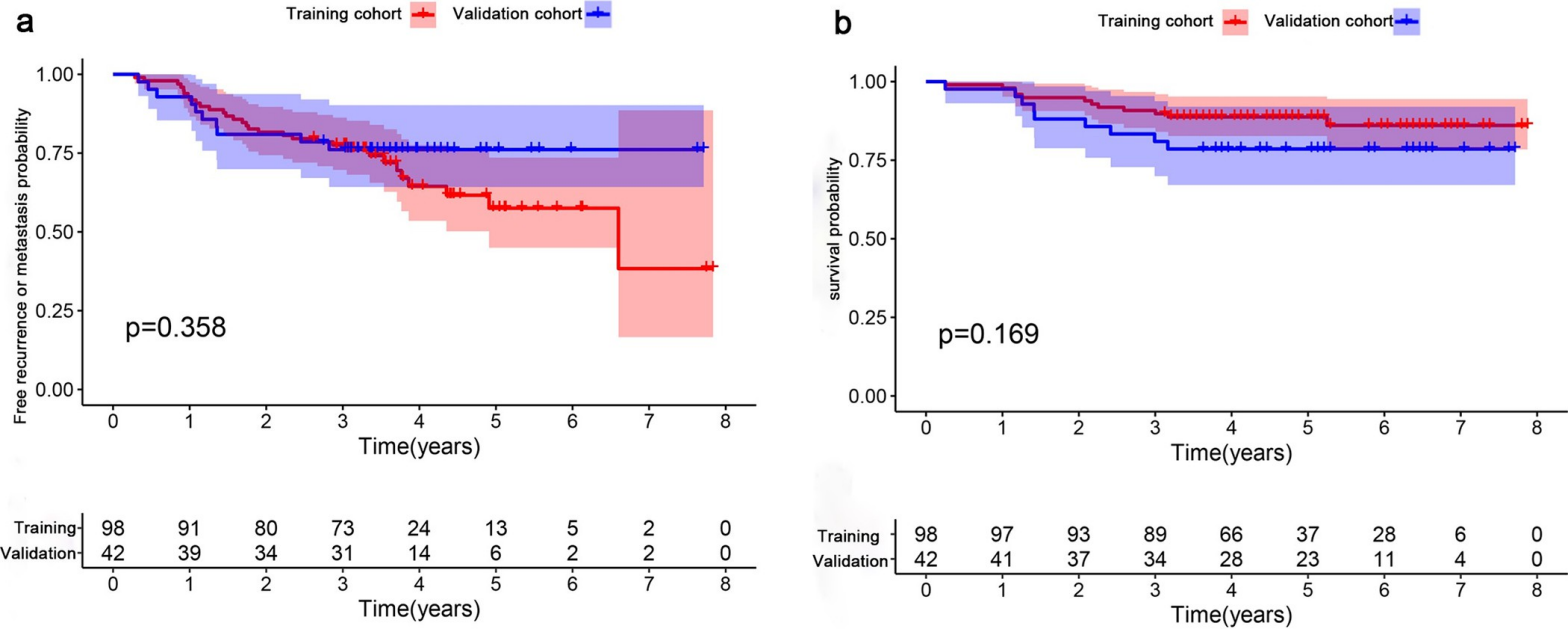
KM curves of training cohort and validation cohort. a) The KM curves of patients with the RMFS, 3-year RMFR were 74.5% and 73.8% in training and validation cohorts, respectively (p>0.05); b) The KM curves of patient’s CSS in training cohort and validation cohort. The 3-year CSS rates were 90.8% and 81%, respectively (p>0.05). P≥0.05 indicates no statistically significant difference between the two groups.

A total of 227 confirmed cases of NPC were collected in this study, and 140 patients were finally enrolled. (Patient screening flow chart is shown in **S1 Fig**). Median follow-up time of 140 patients was 61.36 months (range, 56.89–65.85; 41 patients had recurrence or metastasis, and 99 patients did not). There were no significant differences in sex, stage, treatment scheme and the number of people infected with EBV between the training cohort and the validation cohort. The 3-year RMRF of the training cohort and the validation cohort were 74.5% and 73.8%, respectively (P > 0.05). 3-year CSS was 90.8% in the training cohort and 81% in the validation cohort (P > 0.05). The characteristics of these patients are shown in **Table 1** and **Fig 2** [27–29].

**Table 1:** Baseline tables for the training cohort and validation cohort.

	Training cohort	Validation cohort	*p*		Training cohort	Validation cohort	*P*
	
	98	42			98	42	
**Age(years)**			0.712	>4.73	25(25.5%)	12(26.4%)	
≤47	48.9(49%)	22(52.4%)		**Eosinophils, ×10** ^**9**^ **/L**			0.461
>47	51.0(51%)	20(47.6%)		median (IQR) 0.135 (0.07–0.21)			
**Sex**			0.937	≤0.13	47(48.0%)	23(54.8%)	
man	67(68.4%)	29(69.0%)		>0.13	51(52.0%)	19(45.2%)	
women	31(31.6%)	13(31.0%)		**Basophilic, ×10** ^**9**^ **/L**			0.732
**EBV-DNA**			0.955	median (IQR) 0.02 (0–0.04)			
positive	35(35.7%)	14(33.3%)		≤0.02	60(61.2%)	27(64.3%)	
negative	51(52.1%)	23(54.8%)		>0.02	38(38.8%)	15(35.7%)	
unmeasured	12(12.2%)	5(11.9%)		**Platelet, ×109/L**			
**Nation**			0.535	median (IQR) 227 (194–267)			0.07
ethnic Han	85(86.7%)	38(90.5%)		≤217	47(47.5%)	13(31.0%)	
minority	13(13.3%)	4(9.5%)		>217	52(52.5%)	29(69.0%)	
**Family history**			0.766	**Serum creatinine, umol/L**			
yes	10(10.2%)	5(11.9%)		median (IQR) 71 (60–79)			0.427
no	88(89.8%)	37(88.1%)		≤60	24(24.5%)	13(31.0%)	
**T stage**			0.461	>60	74(75.5%)	29(69.0%)	
T1\T2	51(52.0%)	19(45.2%)		**Blood urea nitrogen, mmol/L**			
T3\T4	47(47.9%)	23(54.8%)		median (IQR) 4.42 (3.55–5.7)			0.138
**N stage**			0.97	≤5	57(58.2%)	30(71.4%)	
N0\N1	40(40.8%)	17(40.5%)		>5	41(41.8%)	12(28.6%)	
N2\N3	58(59.2%)	25(59.5%)		**Albumin, g/L**			
**Induction chemotherapy**			0.283	median (IQR) 40.4 (38–54.3)			0.282
yes	93(94.9%)	37(88.1%)		≤40	51(52.0%)	26(61.9%)	
no	5(5.1%)	5(11.9%)		>40	47(48.0%)	16(38.1%)	
**Adjuvant chemotherapy**			0.891	**Prealbumin, mg/L**			
yes	20(20.4%)	9(21.4%)		median (IQR) 255 (213–292.5)			0.937
no	78(79.6%)	33(78.6%)		≤255	51(52.1%)	20(47.6%)	
**Concurrent chemoradiotherapy**			1	>255	47(47.9%)	22(52.4%)	
yes	90(91.8%)	39(92.9%)		**Alanine aminotransferase**			
no	8(8.2%)	3(7.1%)		median (IQR) 18.5 (14–30)			0.843
**The first blood sample after diagnosis**				≤19	61(68.4%)	28(67.9%)	
**Red blood cell, ×10** ^**12**^ **/L**				>19	37(31.6%)	14(32.1%)	
median (IQR) 4.66 (4.35–4.96)			0.478	**Alkaline phospholipase, U/L**			0.902
≤5.1	84(85.7%)	34(80.9%)		median (IQR) 83 (67.75–94)			
>5.1	14(14.3%)	8(19.1%)		≤92	71(72.4%)	30(71.4%)	
**Hemoglobin, g/L**				>92	27(27.6%)	12(28.6%)	
median (IQR) 141 (129.75–150)			0.483	**Aspartate aminotransferase, U/L**			0.112
≤140	45(45.9%)	22(54.2%)		median (IQR) 22 (19–28)			
>140	84(85.8%)	20(74.6%)		≤24	56(57.1%)	30(71.4%)	
**White blood cell, ×10** ^**9**^ **/L**				>24	42(42.9%)	12(28.6%)	
median (IQR) 6.035 (4.99–7.38)			0.683	**Survival state**			0.169
≤7.13	69(70.4%)	31(73.8%)		live	86(87.8%)	33(78.6%)	
>7.13	29(29.6%)	11(26.2%)		dead	12(12.2%)	9(21.4%)	
**Neutrophil, ×10** ^**9**^ **/L**			0.707	**Recurrence or metastasis state**			0.358
median (IQR) 3.73 (2.99–4.78)				no	67(68.4%)	32(76.2%)	
≤4.73	73(74.5%)	30(73.6%)		yes	31(31.6%)	10(23.9%)	

P-value <0.05 indicated a significant difference. Both cohorts were well balanced in baseline demographic and clinical characteristics. Statistical comparisons between the training and validation cohorts were computed using the χ2 test for categorical variables.

### 3.2 Construction of the CM

The CM was constructed only from the clinical factors (24 clinical features, such as N stage, sex) of NPC. These results showed that PALB, N stage and alanine aminotransferase (AST) level were independent prognostic factors for recurrence or metastasis (**Table 2**) by the Cox univariate and multivariate analysis. Among them, PALB was a protective factor. The risk of N2/N3 was 4 times higher than that of N1/N0 patients. the risk of T3/T4 is 2.23 times higher than that of T1/T2 patients. T stage, N stage, PALB and AST were included in the subsequent construction of the CRM. However, EBV-DNA and adjuvant chemotherapy do not affect recurrence or metastasis in NPC (p > 0.05).

**Table 2:** The Cox univariate and multivariate analysis for clinical characteristics.

	Univariate analysis				Multivariate analysis			
id	HR	HR.95L	HR.95H	pvalue	coef	HR.95L	HR.95H	pvalue
PALB	0.989	0.982	0.995	**0.001**	-0.010	0.982	0.997	**0.009**
N stage (N2/N3 vs. N0/N1)	4.016	1.779	9.069	**0.001**	1.115	1.226	7.587	**0.016**
T stage (T3/T4 vs. T1/T2)	2.230	1.169	4.254	**0.015**	-	-	-	-
AST	1.018	1.002	1.034	**0.023**	0.015	1.000	1.030	**0.049**
PLT	0.995	0.989	1.000	0.071	-	-	-	-
Neutrophil	1.169	0.969	1.411	0.102	-	-	-	-
sex (female vs. male)	0.555	0.265	1.164	0.119	-	-	-	-
adjuvant (yes vs. no)	0.537	0.209	1.377	0.195	-	-	-	-
age	1.016	0.990	1.044	0.231	-	-	-	-
WBC	1.099	0.941	1.285	0.234	-	-	-	-
AlB	0.953	0.874	1.039	0.275	-	-	-	-
basophilic	0.003	0.000	534.878	0.348	-	-	-	-
APL	1.007	0.992	1.022	0.358	-	-	-	-
RBC	0.744	0.382	1.449	0.384	-	-	-	-
crearinine	0.990	0.968	1.013	0.412	-	-	-	-
HB	0.993	0.973	1.014	0.515	-	-	-	-
family history (yes vs. no)	1.333	0.474	3.746	0.586	-	-	-	-
nation (minority vs. Han)	0.789	0.281	2.218	0.653	-	-	-	-
EBV-DNA (positive vs. negative)	1.112	0.563	2.200	0.759	-	-	-	-
ALT	0.997	0.974	1.020	0.769	-	-	-	-
BUN	1.027	0.825	1.280	0.810	-	-	-	-
Eosnophils	0.923	0.131	6.490	0.936	-	-	-	-

P-value <0.05 indicated a significant difference. N stage and AST were regarded as independent prognostic factors. HR, Hazard ratio. CI,confidence interval.

### 3.3 Construction of the RM

Feature extraction and filtering: A total of 960 features were extracted from head and neck enhanced-MRI on PyRadiomics platform by python, including six types (**Fig 1B**). The all features are described in S1 and S2 Tables. including 14 3D shapes, 242 Glcms, 154 Gldms, 176 Glrlms, 176 Glszms and 198 first order. The values of all features were normalized and limited to between 0 and 1 to reduce the variability of feature values (the method is described in S1 File). A total of 773 radiomics features had a good reliability with ICC > 0.75. In order to avoid model overfitting, after filtering out many features by PCC **(Fig 1C)**, 25 and 26 imaging features respectively were selected by RF by using time and state of recurrence or metastasis as endpoints, respectively (**Fig 1C**). Forty-six features were selected, including wavelet-HLH_firstorder_Maximum, wavelet-LHL_glcm_ClusterProminence and so on, which were the union of two results obtained through RF.

Eleven features were associated with recurrence or metastasis finally by Cox univariate analysis. These eleven features were subjected by Cox multivariate regression analysis to construct a RM. Six features were independent prognostic factors. Among them, the features, original_glcm_Idmn and log_sigma_5_0_mm_3D_glszm_ SizeZoneNonUniformityNormalized, had an extremely negative impact on recurrence or metastasis (**Table 3)**.

**Table 3:** The Cox univariate and multivariate analysis for imaging factors.

		Univariate analysis				Multivariate analysis			
id	HR	95L	95H	pvalue	coef		95L	95H	pvalue
log_sigma_3_0_mm_3D_firstorder_10Percentile	5.815	1.195	28.286	**0.029**	-4.352		2.193E-04	0.757	**0.036**
log_sigma_5_0_mm_3D_firstorder_Kurtosis	5.161	1.385	19.229	**0.014**	-		-	-	-
log_sigma_5_0_mm_3D_glszm_SZNUN	0.038	0.004	0.378	**0.005**	-9.358		1.791E-07	0.042	**0.003**
original_glcm_Idmn	209.697	5.263	8355.602	**0.004**	8.065		2.232	4.532E+06	**0.030**
wavelet_HHH_glrlm_LRLGLE	0.025	0.001	0.760	**0.034**	-		-	-	-
wavelet_HHL_firstorder_Maximum	4.759	1.076	21.054	**0.040**	-		-	-	-
wavelet_LHH_firstorder_Mean	0.066	0.005	0.783	**0.031**	-6.272		0.000	0.325	**0.017**
wavelet_LHH_firstorder_Skewness	0.055	0.006	0.518	**0.011**	-5.074		0.000	0.188	**0.003**
wavelet_LHL_firstorder_Skewness	0.030	0.002	0.431	**0.010**	-4.881		0.000	0.198	**0.003**
wavelet_LLH_firstorder_Median	32.487	2.854	369.791	**0.005**	-		-	-	-
wavelet_LLH_glcm_Autocorrelation	4.966	1.171	21.054	**0.030**	1.503		0.813	24.875	0.085

P-value <0.05 indicated a significant difference. eleven radiomic features were related to recurrence or metastasis, including 6 independent prognostic features. HR, Hazard ratio. CI, confidence interval. SZNUN, SizeZoneNonUniformityNormalized. LRLGLE, LongRunLowGrayLevelEmphasis.

### 3.4 Construction of the CRM

It can be seen from the above results that four clinical factors and eleven imaging features were statistically different by Cox univariate analysis. We then constructed a CRM consisting of nine features (seven imaging features and two clinical factors) by Cox multivariate regression analysis. We calculated the Rad-score for each patient using the formula resulting from the 9 features weighted by their regression coefficients as follows: Risk score = —(3.977 × *wavelet_LHH_firstorder_Skewness*)—(4.794 × *log_sigma_3_0_mm_3D_firstorder_10Percentile*)–(5.583 × *wavelet-LHL_firstorder_Skewness*)—(9.460 × *log-sigma-5-0-mm-3D_glszm_SizeZoneNonUniformityNormalized*) + (6.459 × *original_glcm_Idmn*) + (1.462 × *wavelet_LLH_glcm_Autocorrelation*)—(6.665× *wavelet-LHH_firstorder_Mean*) + 0.726 × *N2/N3*–0.01 × *PALB*. Based on median Rad-score, 68 patients were classified as high-risk group (Rad-score >2.03) and 72 as low-risk group (Rad-score ≤2.03).

### 3.5 Evaluation and validation of models

**Fig 3:**
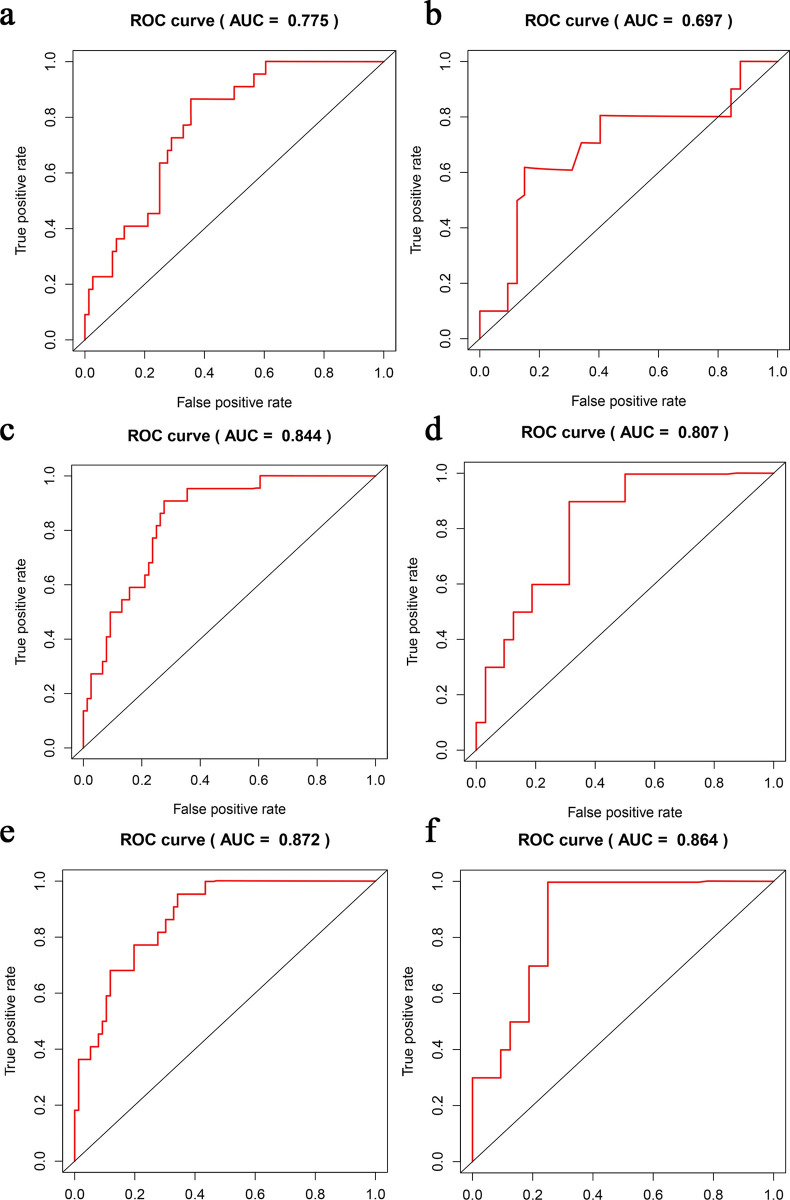
ROCs of the three models. a), c) and e) were the training cohort; b), d) and f) were the validation cohort; a) and b) were the ROCs of the CM, with AUCs of 0.775 (p < 0.001, 95% CI: 0.683–0.867) and 0.697 (p = 0.06,95% CI: 0.498–0.896), respectively; c) and d) ROCs of the RM, with AUCs of 0.844 (p < 0.001, 95% CI, 0.764–0.924) and 0.807 (p = 0.003,95% CI, 0.672–0.942), respectively; e) and f) ROCs of the CRM, with AUCs of 0.872 (p<0.001,95% CI, 0.805–0.939) and 0.864 (p = 0.001,95% CI, 0.756–0.972), respectively.

The median Rad-score divided patients into high- and low-risk groups. The AUCs of the training cohort and validation cohort based on CM were 0.775 (p < 0.001, 95% CI, 0.683–0.867) and 0.697 (p = 0.06, 95% CI, 0.498–0.896), respectively. the sensitivities were 0.742 and 0.600, and the specificities were 0.672 and 0.844, respectively (**Fig 3A and 3B**); The AUCs of the training cohort and validation cohort based on RM were 0.844 (p < 0.001, 95% CI, 0.764–0.924) and 0.807 (p = 0.003,95% CI, 0.672–0.942), respectively. the sensitivities were 0.903 and 0.900, and the specificities were 0.672 and 0.687, respectively (**Fig 3C and 3D**), respectively. Based on CRM, the training cohort’s AUC was 0.872 (p<0.001,95% CI, 0.805–0.939), the sensitivity and specificity were 0.935 and 0.672; the validation cohort’s AUC was 0.864 (p = 0.001, 95% CI, 0.756–0.972), the sensitivity and specificity were 1 and 0.75, respectively. The CRM showed a very good predictive power (**Fig 3E and 3F**). The DeLong test showed the statistical significance between the CM and the CRM in the training cohort (p<0.05). There was no significant difference between the AUC of the combined RM and CRM in the training and validation cohorts (DeLong test).

**Fig 4:**
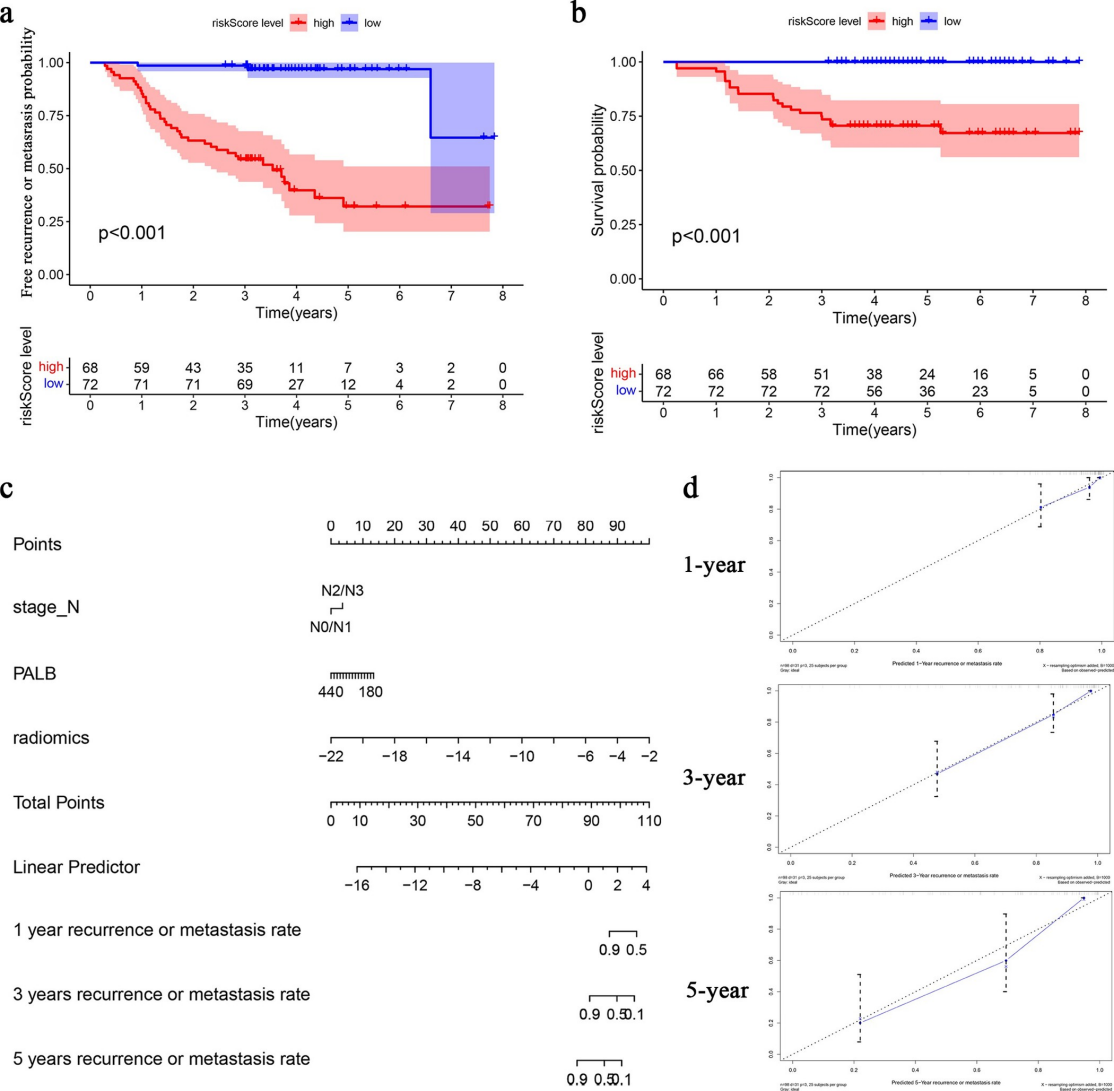
Evaluation and validation of the CRM. a) Kaplan-Meier survival curve for 3-year RMFR. the RMFR in the high-risk group was significantly lower than that in the low-risk group (54.5% vs 96.2%, P<0.001); b) Kaplan-Meier survival curve for the 3-year CSS rates. The 3-year CSS rates were 75% for the high-risk (P <0.001); c) Nomogram predicted the 1-, 3- and 5-year RMFRs of patients with NPC; d) the calibration curves of the 1-, 3-, 5-year recurrence or metastasis, respectively (RM, recurrence or metastasis).

Most NPC recurrence or metastasis within 3 years. The 3-year RMFR were 54.5% in the high-risk group and 96.2% in the low-risk group (p <0.001) (**Fig 4A**), and the 3-year cancer specific survival (CSS) rates were 75% in the high-risk group (p<0.001) (**Fig 4B**). A nomogram was generated based on CRM **(Fig 4C**). Calibration curves showed good fitness for the CRM (**Fig 4D**) that can accurately predict the prognosis of NPC.

### 3.6 For different treatment schemes, the prediction performance of CRM

**Fig 5:**
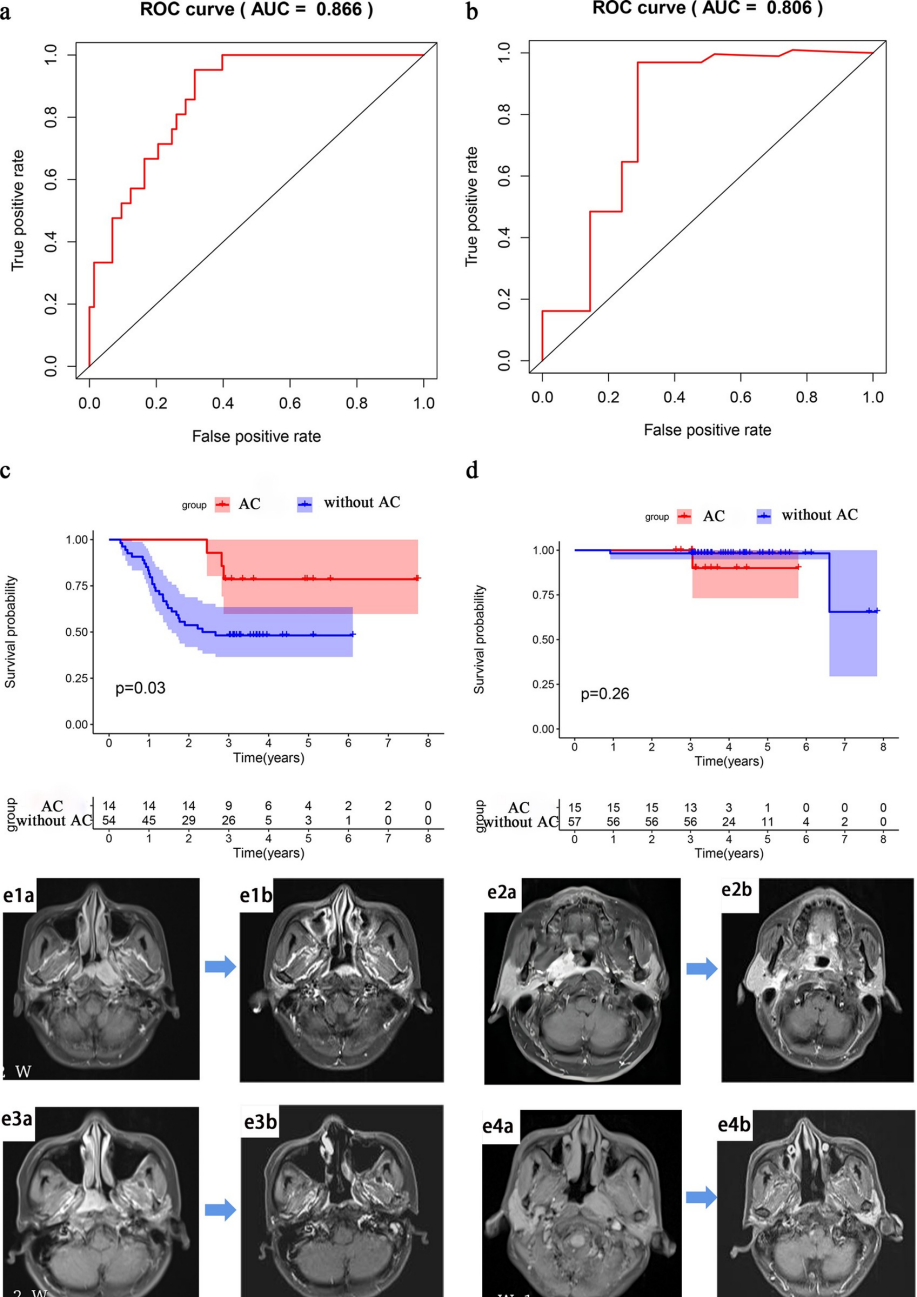
Subgroup analysis of the CRM. a) The group of the IC + CCRT scheme (AUC = 0.866 P<0.001); b) The group of the IC + CCRT + AC scheme (AUC = 0.806 P = 0.013). c) In the high-risk group, the 3-year RMFR of patients receiving AC was significantly higher than the 3-year RMFR of patients not receiving adjuvant chemotherapy (P = 0.03); d) In the low-risk group, the 3-year RMFRs were not statistically significant (P = 0.26); e) Example, In some high-risk patients, e1a), e2a), e3a), e4a) The MRI at initial diagnosis; e1b), e2b) Recurrence was detected by MRI within 3 years of treatment in patients who did not receive adjuvant chemotherapy; e3b), e4b) No recurrence was detected by MRI within 3 years of treatment in patients who received adjuvant chemotherapy.

For LA-NPC, the treatment schemes vary from place to place, especially whether to add AC. To further determine whether the CRM was suitable for the induction with concurrent chemoradiotherapy (IC+CCRT) and the induction, concurrent chemoradiotherapy and adjuvant chemotherapy (IC+CCRT+AC), we calculated AUCs for different patients who received IC + CCRT only, patients who received IC + CCRT + AC, and all enrolled patients. We found that the CRM predicted the 3-year RMFR of patients with IC+CCRT with an AUC of 0.866 (p<0.001, 95% CI, 0.795–0.936); At the same time, in IC+CCRT+AC group, the AUC was 0.806 (p = 0.013, 95% CI, 0.645–0.936) (**Fig 5A and 5B**), and high sensitivity and specificity were achieved in both groups (**Table 4)**. The model had high accuracy for both treatment schemes.

**Table 4:** The AUC values, sensitivity and specificity of the different treatment modes in CRM.

	AUC (95% CI)	P value	sensitive	specificity
IC+CCRT	0.866 (0.795–0.936)	<0.001	0.926	0.801
IC+CCRT+AC	0.806 (0.644–0.968)	0.013	1.000	0.632
all patients	0.869 (0.812–0.926)	<0.001	0.951	0.687

P-value <0.05 indicated a significant difference. IC+CCRT, induction combined with concurrent chemoradiotherapy; IC+CCRT+AC: induction, concurrent chemoradiotherapy and adjuvant chemotherapy.

### 3.7 Only the high-risk patients in the CMR were recommended adjuvant chemotherapy (AC)

Not all patients with NPC are suitable for AC. After adding AC to some patients, the toxicity and cost increased, and there was no obvious survival benefit [5, 30]. Therefore, it is necessary to explore which patients are suitable for AC. We explored the efficacy of AC in patients in the high- and low-risk groups respectively. The results showed that in the high-risk group, the 3-year RMFR of patients who received AC was 78.6%, and the 3-year RMFR of patients who did not receive AC was only 48.1% (p = 0.03). In the low-risk group, the 3-year RMFR was not significant difference (p = 0.26) **(Fig 5C and 5D**). It was suggested that for high-risk patients, the combination of AC may should be recommended. For low-risk patients, AC was not recommended because of no benefit and increased toxicity. The model was able to filter patients who would benefit from AC. (Examples: in some high-risk patients, no recurrence or metastasis were found after AC, and recurrence was found after no AC **Fig 5E**)

## 4. Discussion

In fact, AC remains a controversial treatment because previous study failed to demonstrate clinical effectiveness [5, 31]. Chan et al. found that, in patients with NPC with detectable post-RT plasma EBV DNA, AC with cisplatin and gemcitabine did not improve relapse-free survival (49.3% vs. 54.7%; P = 0.75) [31]. However, Tao et al. revealed that there were differences between the IC+CCRT and IC+CCRT+ AC groups in terms of the 5-year overall survival (OS) (78.9% vs. 85.0%, P = 0.034), disease-free survival (DFS) (73.4% vs. 81.7%, P = 0.029), and distant metastasis-free survival (DMFS) (84.9% vs. 76.0%, P = 0.019) in N2/3 positive NPC patients [11]. Why the results were opposite? The biggest reason is that many studies did not stratify patients. Hui et al. constructed a risk prediction model to integrate postradiotherapy EBV-DNA and TNM stage for risk stratification of NPC patients after completion of radio-/chemoradiotherapy to AC or observation. The findings showed that AC in low-risk group did not benefit and increases toxicities. But the limitation was that the model could not predict the survival benefits from adjuvant chemotherapy in high-risk group [32]. In addition, Shen et al. found that in the high-risk group derived from radiomic scoring, CCRT+AC achieved significantly better PFS, LRRFS, DMFS and OS than IC+CCRT. In the low-risk group, IC+CCRT yielded significantly better outcomes than CCRT+AC [33]. It is necessary to identify patients who may benefit more from AC to reduce side effects and unnecessary costs. In this study, we constructed a CRM to predict the 3-year RMFR in NPC patients based on MRI radiomics and clinical factors. The CRM could divide patients into the high-risk and low-risk groups. More importantly, AC could improve 3-year RMFR in high-risk patients in CRM.

I compared the predictive performance to previous studies on the same data. It was found that the AUC values of our model were higher in both the training and validation cohort (S2 Fig) [34–36], but these results contain more features and sequences. In our study, the CRM showed higher predictive performance than CM, in both the training and the validation cohorts. Similar to our results, these studies found that MRI radiomics combined with clinical factors has better predictive power than that of traditional TNM staging in predicting the PFS and OS of NPC [37–39]. The main reason is that radiomics transforms the image into data information visible to the naked eye by extracting the features of the internal and surrounding tissues of the tumour, thereby revealing the internal heterogeneity of cancer tissue such as cytology, physiology and genetic informatics [21]. Texture features are mathematical parameters computed from the distribution of pixels, which can represent the heterogeneity of voxel arrangement in the tumour area [40]. The above point confirmed the result that there was no significant difference between the AUC of the RM and CRM in the training and validation cohorts (DeLong test). This was the reason why CM performs well in predicting RMFR in patients [41].

Finally, our CRM included 7 imaging features. Among these factors, we identified a powerful feature, original-glcm-Idmn. Idmn is also called the inverse difference moment normalized, which reflects the homogeneity of the image texture and measures the local change of the image texture. original-glcm-Idmn is a widely extracted texture feature, and may be positively correlated with NPC recurrence or metastasis [19]. Wavelet features reflect tumor information from eight spatial domains, and the “skewness” in wavelet subspace shows that tumor heterogeneity described by entropy and tumor intensity has prognostic value in high dimensional wavelet and logarithmic space [42]. In addition to, skewness was highly important in the RM by the study of Fan. The findings showed that luminal A had lower values of skewness and kurtosis features compared with luminal B in breast cancer. And, among the different prognostic models constructed by different combinations of features and clinical factors, only these two characteristics appeared most frequently [43].

Among the steps in radiomics, the most important steps are feature selection and model building. The limitation is that most feature extraction methods are low-quality and complex, and the results are not convincing. We filter features by PCC and RF and build the model by Cox multivariate regression analysis. The AUCs of the training and validation cohorts of the CRM were 0.872 (p<0.001,95% CI, 0.805–0.939), and 0.864 (p = 0.001,95% CI, 0.756–0.972), respectively. The AUCs were higher than that of the models built by logistic regression after filtering features by other statistical methods such as absolute shrinkage and selection operator (LASSO) (AUC, 0.7–0.8) [44]. RF, as a statistical method with high classification accuracy and efficiency, has high prognostic performance and good stability to data fluctuations. the data dimensionality was reduced by random forest, which is a good filtering method to solve model overfitting [45]. Zhang et al. found that the consistency index (c-index) values of the model constructed by RF screening features were about 0.82 in both internal and external validation cohorts [20]. PCC is the most commonly used statistical tool to reflect the degree of linear correlation between two variables and reduce the factors that influence each other [46]. Li et al. used radiomics to predict the prognosis of cervical cancer and selected the features by the PCC, and the c-index value of the model was also around 0.75 [47]. In addition to LASSO, many studies filtered features such as L1-logistic regression method and built models with support vector machine (SVM), which can also predict the prognosis, and their C-index and AUCs were about 0.7–0.8 [48–50].

The CRM included 2 clinical factors (N stage and PALB). In clinical stage, only N stage was included in the final combination model, excluding T stage. Possibly because T stage represents the size of the tumour and extent of invasion, and has little correlation with the microinvasion of the tumour, which was consistent with the results of some clinical studies [48, 51]. PALB was a positive factor in our study. The reason may be related to nutrition. Li et al. also found a positive correlation between ALB measurements and the overall survival rate [52]. EBV-DNA, as an early screening and prognostic indicator, should theoretically be associated with recurrence or metastasis [53–55]. In addition, Hu et al. found that AC might reduce the incidence of locoregional recurrence or distant metastasis in patients without post-radiotherapy plasma EBV DNA clearance [32]. However, in this study, EBV-DNA was not a predictive factor, which may be because thirty percent of the patients were not tested for EBV-DNA or because we performed only qualitative testing instead of quantitative testing.

In a 2012 study, Chen et al. found that AC may only increase toxicity. However, in recent years, they have also found that adjuvant capecitabine could improve outcomes in a specific population [5, 56]. Many studies found that the addition of AC in N2/3 patients can further improve the OS, DFS, and PFS, and the side effects were acceptable [11, 12]. Therefore, we need to stratify patients with NPC and look for patients who can benefit from AC. Several studies have accurately predicted the prognosis of IC by radiomics and selected patients suitable for induction chemotherapy [57–59]. Similarly, Keek et al. predicted the risk of local recurrence and distant metastases after CCRT by radiomics for patients with squamous carcinoma of the head and neck [60]. However, the most controversial aspect of AC has been less studied. Our final model accurately stratified NPC patients by radiomics and clinical factors. We found that in the high-risk group (Rad-score >2.03), the 3-year RMFR of patients receiving AC was significantly higher than that of patients without AC (p = 0.03), suggesting that for high-risk patients, combined AC was more recommended. In the low-risk group, the result showed that there was no statistical difference in 3-year RMFR regardless of whether they received AC or not. But for low-risk patients who received AC, the cumulative effect of chemotherapy lead to increased side effects (such as stomatitis, leucopenia) [5], decreased quality of life, and increased cost-effectiveness [30], which may suggest that combined AC was not applicable for low-risk patients [5].

Our study also had some limitations. First, only the phase with the most obvious enhanced MRI was selected for analysis. Further requests should be made for T1WI, T2WI and DWI images. Second, the metastatic lymph nodes are also an important prognostic factor in NPC. Therefore, prognosis may be more accurately predicted by extracting metastatic lymph node region (GTVnd) and gross tumor volume (GTV). Third, we lacked external validation. Further external validation is required to improve diagnostic accuracy, sensitivity and specificity before clinical application of CRM [61, 62].

## 5. Conclusion

In conclusion, the accurate predictive model provided a noninvasive way to predict the outcomes of NPC and helped identify high-risk patients who benefited from AC for improve the 3-year RMFR. AC might not be necessary for low-risk patients.

## Supporting information

S1 Fig Patient screening flow chart. (PNG)

S2 Fig Comparison of AUC values between other studies and our study ROC curves. (TIF)

S1 Table Features extracted from targets outlined by an associate chief physician. (XLSX)

S2 Table Features extracted from targets outlined by another associate chief physician. (XLSX)

S1 File Other supplementary materials. (DOCX)

S2 File Supplementary material_code. (DOCX)

## Abbreviations

AUC: area under curve
AC: adjuvant chemotherapy
C-index: consistency index
CM: clinical model
CRM: clinical-radiomics model
CSS: cancer specific survival
GTV: gross tumour volume
GTVnd: lymph node gross tumour volume
ICC: intra- and interclass correlation coefficient
IC+CCRT: induction with concurrent chemoradiotherapy
IC+CCRT+AC: induction, concurrent chemoradiotherapy and adjuvant chemotherapy
LASSO: least absolute shrinkage and selection operator
NPC: nasopharyngeal carcinoma
PCC: pearson correlation coefficient
RF: random forest
RM: radiomics model
RMFR: recurrence or metastasis free rate
RMFS: recurrence or metastasis free survival
