# Supplementary figures and images for: Adjuvant chemotherapy or no adjuvant chemotherapy? A prediction model for the risk stratification of recurrence or metastasis of nasopharyngeal carcinoma combining MRI radiomics with clinical factors
Source: PLoS One. 2023 Sep 26;18(9):e0287031. doi: 10.1371/journal.pone.0287031 (PMC10522047; doi:10.1371/journal.pone.0287031)

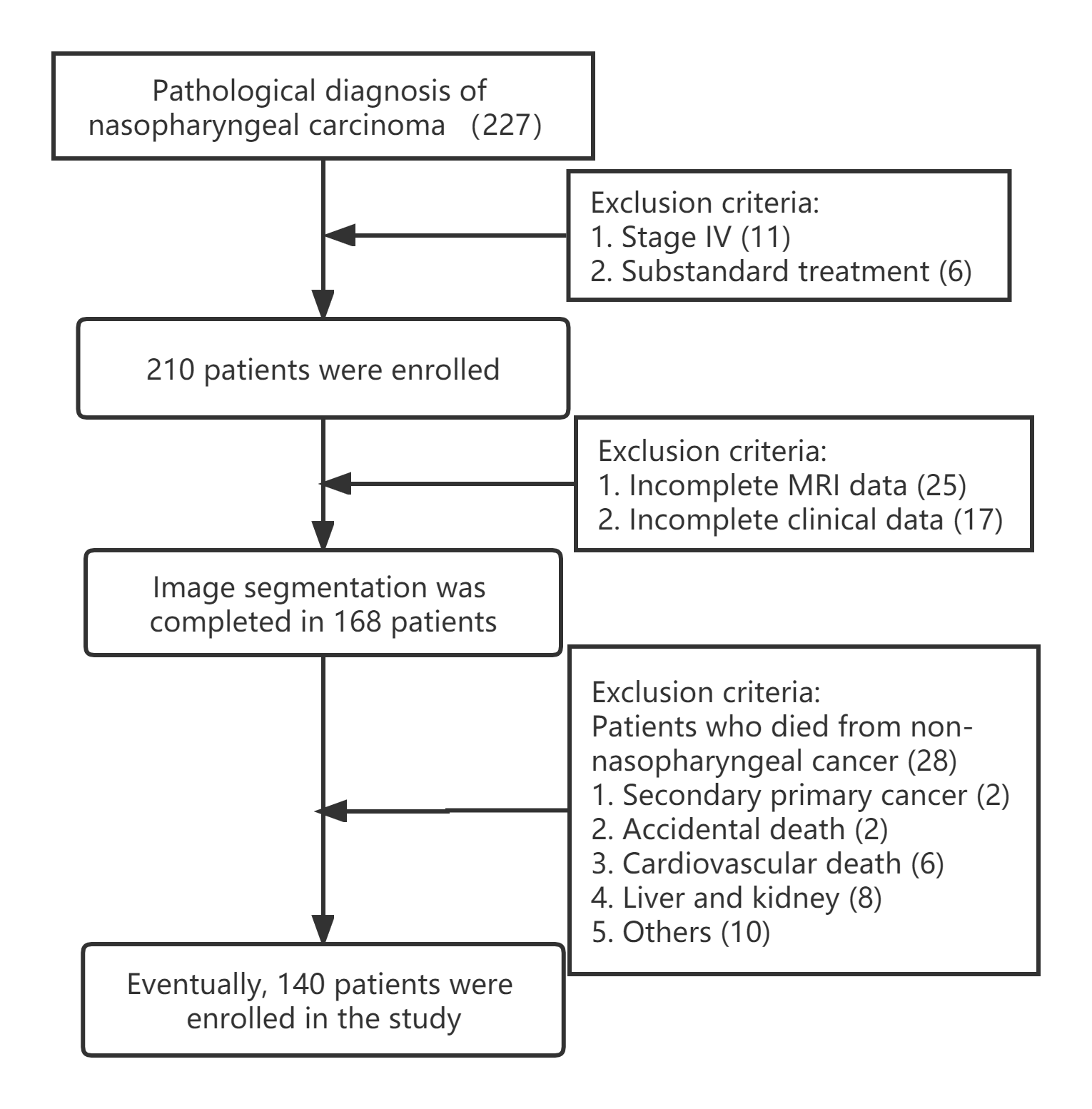

Supplement: S1 Fig — (PNG) [file pone.0287031.s001.png]

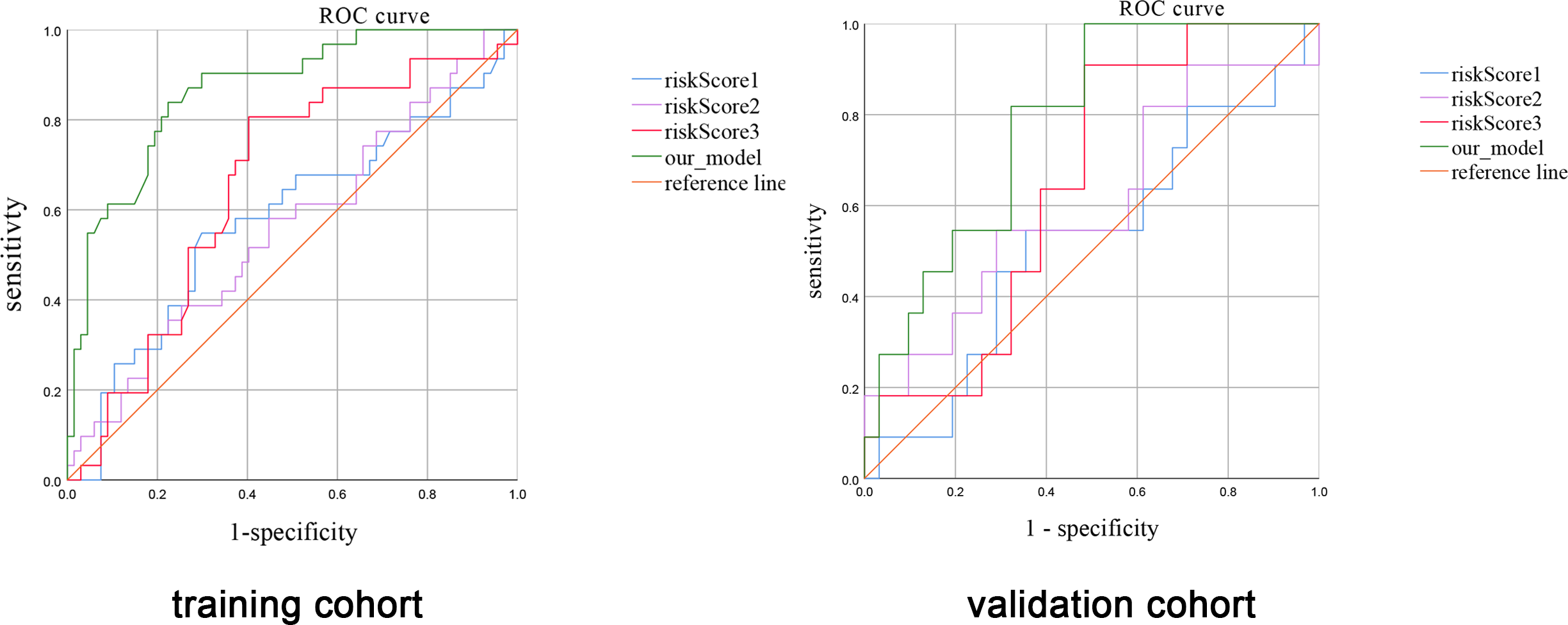

Supplement: S2 Fig — (TIF) [file pone.0287031.s002.tif]
