# Supplementary material for: Adjuvant chemotherapy or no adjuvant chemotherapy? A prediction model for the risk stratification of recurrence or metastasis of nasopharyngeal carcinoma combining MRI radiomics with clinical factors
Source: PLoS One. 2023 Sep 26;18(9):e0287031. doi: 10.1371/journal.pone.0287031 (PMC10522047; doi:10.1371/journal.pone.0287031)
Supplement: S1 File — (DOCX) [file pone.0287031.s005.docx]

**Supplementary Information**

**radiomics feature extraction**

MRI scan parameters are as follows：TR:350ms, TE:10ms, FoV: 250mm*250mm，thickness: 5mm, space:1mm. Features were extracted from the planning MRI using the Python “pyradiomics” package (version 3.6). Resampling the image with nearest neighbor method (3.0*3.0*3.0mm). BinWidth: 5. The filtered image is generated by using all combinations of high-pass and xiow-pass filters (LLH, LHL, LHH, HLL, HLH, HHL, HHL, HHH, LLL) for the original image in the x-/y-/z-direction, respectively. In addition, The Laplacian of Gaussian (LoG) filter was also applied to the original image, and the corresponding image was derived for each sigma. The sigma value was specified as 3.0 mm and 5.0 mm. In my study, 960 radiomics features were extracted, including 14 shapes, 242 Glcms, 154 Gldms, 176 Glrlms, 176 Glszms and 198 first order. PyRadiomics was developed with Image Biomarker Standardization Initiative (IBSI) in mind, but some of the extracted features are defined with subtle differences. Among the differences, we chose IBSI. The Feature extraction was standardized according to the IBSI. Good repeatability features filtered by ICC (>0.75).

All extracted features were normalized:

X' = (X - X_min_) / (X_max_ - X_min_)

X is the original feature value, X' is the normalized feature value, X_max_ is the maximum value of the feature, X_min_ is the minimum value of the feature.

The normalized data were filtered for features by PCC, random forest, and Cox univariate and multivariate analysis to construct the model.

*Abbreviations:* TR: repetition time; TE: echo time; FOV: field of view

**Radiomics feature**

**First-order statistics features:** First-order statistics describe the distribution of voxel intensities within the MRI image through commonly used and basic metrics. Eighteen first-order statistics features were used, such as energy, entropy, skewness, kurtosis, mean, maximum, and minimum.

**Shape features (2D, 3D):** The features included descriptors of the three-, two-dimensional size and shape of the ROI. Fourteen features were used, such as MeshVolume, SurfaceVolumeRatio, sphericity.

**Wavelet features**

The filtered image is generated by using all combinations of high-pass and low-pass filters (LLH, LHL, LHH, HLL, HLH, HHL, HHL, HHH, LLL) for the original image in the x-/y-/z-direction, respectively. For each decomposition, In the end, we extracted 689 Wavelet features for each of the MRI series.

**Laplacian of Gaussian filter** (LoG)

LoG was also applied to the original image, and the corresponding image was derived for each sigma. Is is an edge enhancement filter, with low sigma emphasizing fine textures and high sigma values emphasizing coarse textures. The sigma value was specified as 3 mm and 5 mm in this study in our study.

**Statistics-based textural features:**

In the Gray Level Co-occurrence Matrix (GLCM), matrix describes the number of times the combination of voxels with grey level i and j appear in the ROI. Gray-level run length metrics (GLRLM) is defined as the length of consecutive pixels with the same gray scale value. A Gray Level Size Zone (GLSZM) quantifies gray level zones in an image. The GLSZM is defined as the number of connected voxels that share the same gray level intensity. A Gray Level Dependence Matrix (GLDM) quantifies gray level dependencies in an image. A gray level dependency is defined as the number of connected voxels within distance δ that are dependent on the center voxel. And, A Gray Level Run Length Matrix (GLRLM) quantifies gray level runs, which are defined as the length in number of pixels, of consecutive pixels that have the same gray level value. We extracted 22 radiomics features from the GLCM, 16 features from the GLSZM, 14 feature from GLDM, and 16 features from the GLRLM.

**Compared the predictive performance to previous studies on the same problem/data**

**
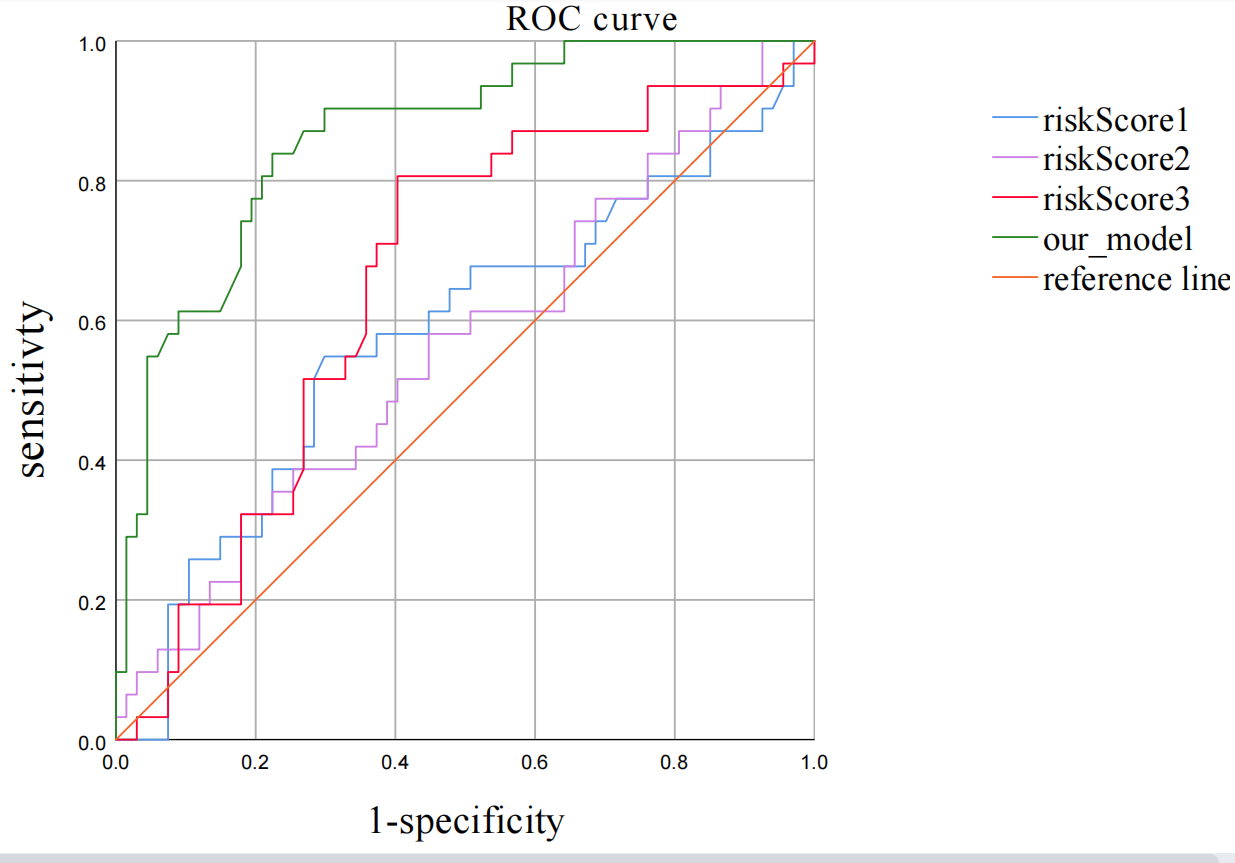
**

**Train cohort**

**
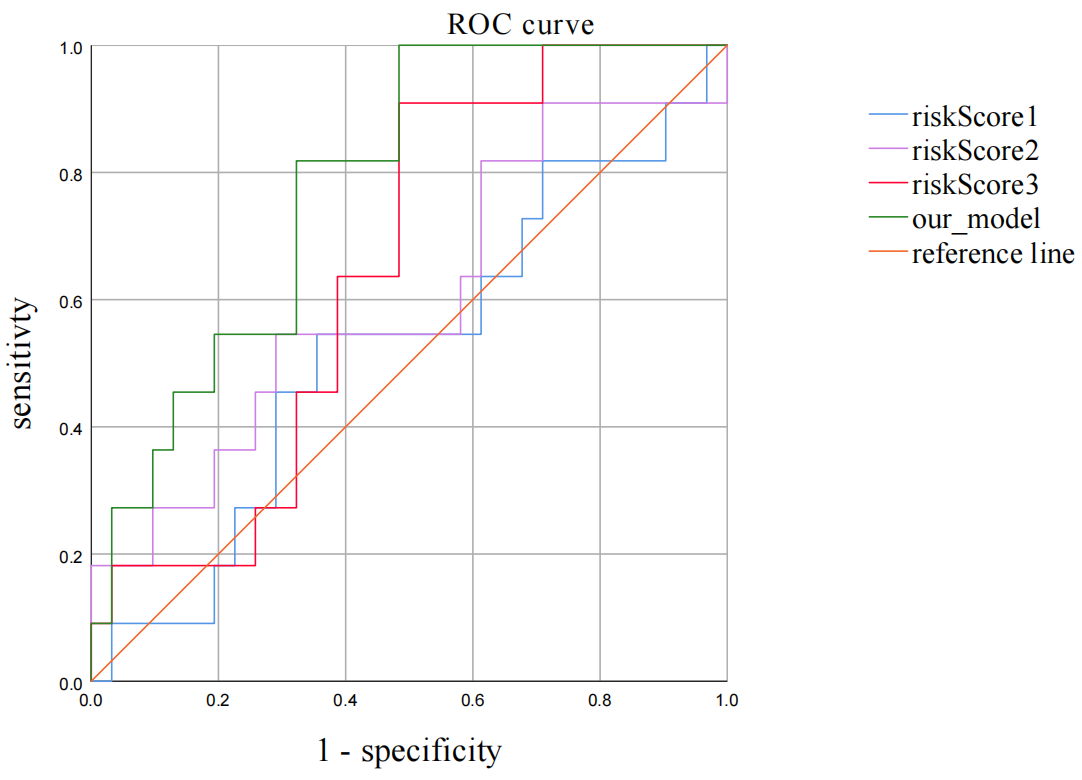
Test cohort**

riskScore1 (doi: 10.1002/hed.26867), riskScore2 (doi.org/10.1007/s12672-021-00460-3), riskScore3 (doi:10.7150/jca.33345)
