# Supplementary material for: Adjuvant chemotherapy or no adjuvant chemotherapy? A prediction model for the risk stratification of recurrence or metastasis of nasopharyngeal carcinoma combining MRI radiomics with clinical factors
Source: PLoS One. 2023 Sep 26;18(9):e0287031. doi: 10.1371/journal.pone.0287031 (PMC10522047; doi:10.1371/journal.pone.0287031)
Supplement: S2 File — (DOCX) [file pone.0287031.s006.docx]

{

"cells": [

{

"cell_type": "code",

"execution_count": 8,

"metadata": {},

"outputs": [],

"source": [

"import radiomics\n",

"from radiomics import featureextractor\n",

"import pandas as pd\n",

"import glob\n",

"import os"

]

},

{

"cell_type": "code",

"execution_count": 9,

"metadata": {},

"outputs": [],

"source": [

"data_dir = \"/home/cyh/data/Nasopharyngeal/\"\n",

"data_pairs = os.listdir(data_dir)\n",

"\n",

"df = pd.DataFrame()\n",

"params = \"./settings.yaml\"\n",

"extractor = featureextractor.RadiomicsFeatureExtractor(params)\n",

"\n",

"for data_pair in data_pairs:\n",

" files = os.listdir(os.path.join(data_dir,data_pair))\n",

" if len(files) != 2 :\n",

" print(\"file count err\")\n",

" if files[0].endswith(\".nii\"):\n",

" imageFile = os.path.join(data_dir,data_pair,files[0])\n",

" maskFile = os.path.join(data_dir,data_pair,files[1])\n",

" else:\n",

" imageFile = os.path.join(data_dir,data_pair,files[1])\n",

" maskFile = os.path.join(data_dir,data_pair,files[0])\n",

" featureVector = extractor.execute(imageFile,maskFile)\n",

" df_add = pd.DataFrame.from_dict(featureVector.values()).T\n",

" df_add.columns = featureVector.keys()\n",

" df = pd.concat([df,df_add])\n",

"df.to_excel(\"features.xlsx\")\n"

]

}

],

"metadata": {

"kernelspec": {

"display_name": "sci",

"language": "python",

"name": "python3"

},

"language_info": {

"codemirror_mode": {

"name": "ipython",

"version": 3

},

"file_extension": ".py",

"mimetype": "text/x-python",

"name": "python",

"nbconvert_exporter": "python",

"pygments_lexer": "ipython3",

"version": "3.8.16"

},

"orig_nbformat": 4

},

"nbformat": 4,

"nbformat_minor": 2

}

# This is an example of settings that can be used as a starting point for analyzing MR data with large(r) (~5mm) slice

# thickness. This is only intended as a starting point and is not likely to be the optimal settings for your dataset.

# Some points in determining better values are added as comments where appropriate

# When adapting and using these settings for an analysis, be sure to add the PyRadiomics version used to allow you to

# easily recreate your extraction at a later timepoint:

# ############################# Extracted using PyRadiomics version: <version> ######################################

imageType:

Original: {}

LoG:

# Because of resampling to (3, 3, 3), the use of sigmas < 3 mm is not recommended.

sigma: [3.0, 5.0]

Wavelet: {}

featureClass:

# redundant Compactness 1, Compactness 2 an Spherical Disproportion features are disabled by default, they can be

# enabled by specifying individual feature names (as is done for glcm) and including them in the list.

shape:

firstorder:

glcm: # Disable SumAverage by specifying all other GLCM features available

- 'Autocorrelation'

- 'JointAverage'

- 'ClusterProminence'

- 'ClusterShade'

- 'ClusterTendency'

- 'Contrast'

- 'Correlation'

- 'DifferenceAverage'

- 'DifferenceEntropy'

- 'DifferenceVariance'

- 'JointEnergy'

- 'JointEntropy'

- 'Imc1'

- 'Imc2'

- 'Idm'

- 'Idmn'

- 'Id'

- 'Idn'

- 'InverseVariance'

- 'MaximumProbability'

- 'SumEntropy'

- 'SumSquares'

glrlm:

glszm:

gldm:

setting:

# Normalization:

# MR signal is usually relative, with large differences between scanners and vendors. By normalizing the image before

# feature calculation, this confounding effect may be reduced. However, if only one specific scanner is used, or the

# images reflect some absolute world value (e.g. ADC maps, T2maps (NOT T2 weighted)), consider disabling the

# normalization.

normalize: true

normalizeScale: 100 # This allows you to use more or less the same bin width.

# Resampling:

# Increasing the resampled spacing forces PyRadiomics to look at more coarse textures, which may or

# may not increase accuracy and stability of your extracted features. Using a small spacing in large slices generates

# many interpolated voxels, potentially 'masking' the signal contained in the image.

interpolator: 'sitkBSpline'

resampledPixelSpacing: [3, 3, 3]

# Mask validation:

# correctMask and geometryTolerance are not needed, as both image and mask are resampled, if you expect very small

# masks, consider to enable a size constraint by uncommenting settings below:

#minimumROIDimensions: 2

#minimumROISize: 50

# Image discretization:

# The ideal number of bins is somewhere in the order of 16-128 bins. A possible way to define a good binwidt is to

# extract firstorder:Range from the dataset to analyze, and choose a binwidth so, that range/binwidth remains approximately

# in this range of bins.

binWidth: 5

# first order specific settings:

# When normalizing, gray values below the mean will be negative. Shifting by 300 (3 StdDevs * 100) ensures that the

# majority of voxels is positive (only outliers >3 SD lower than the mean will be negative).

voxelArrayShift: 300

# Misc:

# default label value. Labels can also be defined in the call to featureextractor.execute, as a commandline argument,

# or in a column "Label" in the input csv (batchprocessing)

label: 1
